# Loss of Egr1, a human del5q gene, accelerates BCR-ABL driven chronic myelogenous leukemia

**DOI:** 10.18632/oncotarget.20612

**Published:** 2017-09-01

**Authors:** Silvia Maifrede, Andrew Magimaidas, Xiaojin Sha, Kaushiki Mukherjee, Dan A. Liebermann, Barbara Hoffman

**Affiliations:** ^1^ Fels Institute for Cancer Research and Molecular Biology, Temple University Lewis Katz School of Medicine, Philadelphia, PA, USA; ^2^ Current address: Department of Systems Pharmacology and Translational Therapeutics, Perelman School of Medicine, University of Pennsylvania; ^3^ Department of Microbiology and Immunology, Temple University Lewis Katz School of Medicine, Philadelphia, PA, USA; ^4^ Department of Medical Genetics and Molecular Biochemistry, Temple University Lewis Katz School of Medicine, Philadelphia, PA, USA

**Keywords:** Egr1, chronic myelogenous leukemia, stress response protein, tumor suppressor, Leukemic stem cells

## Abstract

There is substantial evidence that early growth response-1 (Egr1) gene, a zinc-finger transcription factor, behaves as a tumor suppressor in leukemia. This includes reports from this laboratory that constitutive Egr1 overrides leukemia conferred by deregulated c-Myc or E2F-1 in the M1 myeloid leukemic cell line by promoting differentiation. To investigate the effect of Egr1 on the initiation and progression of Chronic Myelogenous Leukemia (CML), lethally irradiated syngeneic wild type mice were reconstituted with bone marrow (BM) from either wild type or Egr1 null mice transduced with a 210-kD BCR-ABL-expressing MSCV-retrovirus (bone marrow transplantation {BMT}). Loss of Egr1 was observed to accelerate the development of BCR-ABL driven leukemia in recipient mice, resulting in the development of a more aggressive disease, a significantly shortened median survival time, and increased BCR-ABL expressing leukemic stem/progenitor cells (GFP+Lin-cKit+Sca+). *Egr1* deficient progenitors expressing BCR-ABL exhibited decreased apoptosis, and increased cell viability and proliferation relative to WT counterparts. Secondary BMT of BCR-ABL BM revealed that loss of *Egr1* resulted in enrichment of LSCs, consistent with shorter survival time and more aggressive disease of these mice compared to WT counterparts. Furthermore, serial re-plating colony assays indicated that loss of *Egr1* increased self-renewal ability of BCR-ABL expressing BM. These novel findings on the tumor suppressor role of Egr1 in CML provide the impetus to study the effect of altering Egr1 expression in AML, where the overall five year survival rate remains low. The effect of loss of Egr1 in CML could reflect its established functions in normal hematopoiesis, maintaining quiescence of HSCs and driving terminal differentiation to the monocyte/macrophage lineage. Gain of function studies should validate these conclusions and provide further rationale for increased Egr1 as a therapeutic target in AML.

## INTRODUCTION

The early growth response-1 (Egr1) gene, a zinc-finger transcription factor localized to the human chromosome 5 [1], is rapidly stimulated by growth factors, hormones and neurotransmitters [2]. In addition, Egr1 is a myeloid differentiation primary response (MYD) gene, and is a positive regulator of terminal myeloid differentiation that potentiates macrophage differentiation [3-8]. There is substantial evidence that Egr1 behaves as a tumor suppressor in leukemia. Deletions in the 5q region, where Egr1 and several other tumor suppressor genes are mapped, is often observed in therapy-related myeloid neoplasms (t-MN) [9], a subset of patients with primary myelodysplastic syndrome (MDS), and about 15% of patients with acute myeloid leukemia (AML) de novo [10]. Furthermore, it has been demonstrated in mouse models that haplo-insufficiency of Egr1 leads to development of myeloid disorders [11, 27-28]. This laboratory has demonstrated that constitutive Egr1 can override leukemia conferred by deregulated c-Myc or E2F-1 in the M1 myeloid leukemic cell line by promoting differentiation [11-13]. It has also been shown that Ezh2 directly regulates Egr1 in hematopoiesis, and that deletion of Ezh2 and the reactivation of genes repressed by it, including Egr1, converted a high-grade myeloid leukemia to a less aggressive myeloid neoplasia (MPN) [14, 15]. Another study has demonstrated that Egr1 can also behave as a tumor suppressor in AML1-ETO-positive AML [16]. In addition, Egr1 directly regulates multiple tumor suppressor genes including TGFβ1, PTEN, p53 and fibronectin [17], some of which have been implicated to regulate CML [18-20]. This large body of evidence consistent with Egr1 behaving as a tumor suppressor in hematopoietic cells, both *in vivo* & *in vitro* and in both humans and mice, led us to ask if Egr1 plays a role in Chronic Myelogenous Leukemia (CML).

CML is a hematological disease originating from a reciprocal chromosomal translocation t(9;22)(q34;q11) in pluripotent hematopoietic stem cells, generating the Philadelphia chromosome (Ph) [21]. This translocation results in the chimeric BCR-ABL oncogene, encoding for a constitutively active protein kinase [22]. CML is typically diagnosed in chronic phase (CP), characterized by elevated levels of granulocytes. If left untreated additional mutations arise that impact on differentiation, DNA repair and telomere maintenance, as well as loss of tumor suppressor genes [23], with concomitant disease progression. There is transition to accelerated phase (AP) and ultimately to blast crisis (BC) associated with rapid expansion of blast cells [23-24].

In this study we used a mouse model of bone marrow transplantation (BMT) for BCR-ABL driven leukemia and observed that loss of *Egr1* in BCR-ABL expressing bone marrow (BM) accelerated the onset of myeloid leukemia. We also observed that *Egr1* expression is down-regulated by BCR-ABL. Furthermore, we observed increased self-renewal ability of BCR-ABL-expressing Egr1 KO BM, which correlated with increased leukemic potential and higher number of leukemia initiating cells. Our studies now demonstrate for the first time that *Egr1* behaves as a tumor suppressor in a mouse model of BCR-ABL driven leukemia, and provides the impetus to study the effect of altered Egr1 in Acute Myelogenous Leukemia (AML) where the overall five year survival rate remains low.

## RESULTS

### Loss of *Egr1* accelerates the onset of BCR-ABL driven leukemia

**Figure 1:**
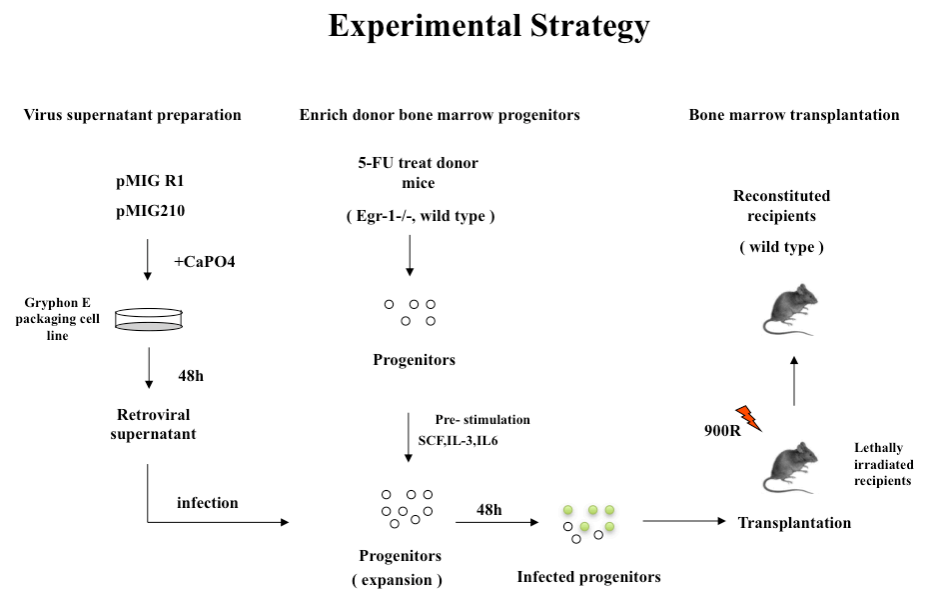
Schematic representation of the experimental strategy used in all BMT experiments described

**Figure 2:**
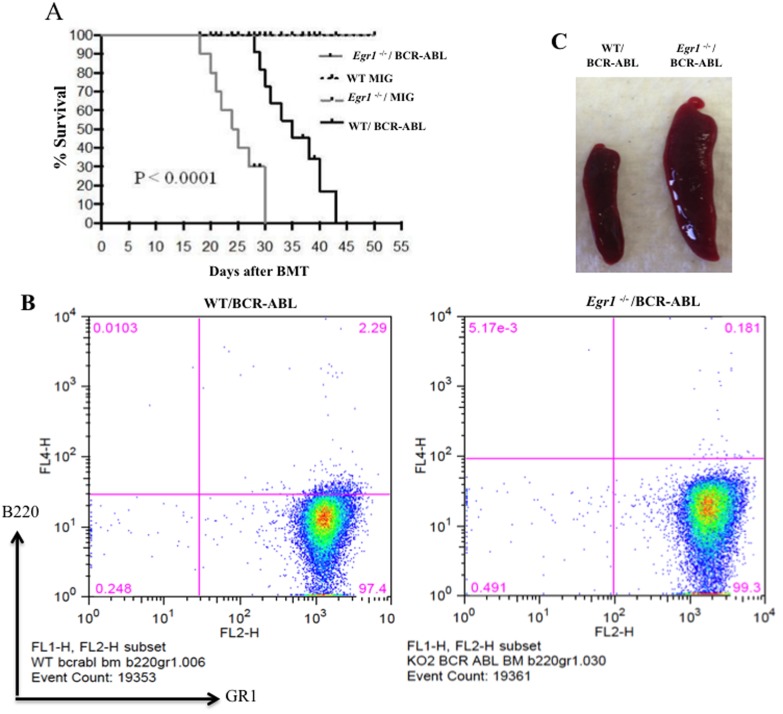
Loss of *Egr1* accelerated CML development in mice **A.** Kaplan-Meier survival curves for recipients of MIGR1 or MIG BCR-ABL-transduced BM cells from WT or *Egr1*^-/-^ donor mice. WT recipient mice were transplanted with BCR-ABL-expressing BM or MIGR1-expressing BM. Following transplantation, animals were observed for signs of disease and euthanized at first signs of morbidity. Statistical analysis used was Log-rank (mantel-Cox) test, P value < 0.0001. *Egr1*^-/-^/BCR-ABL (*n* = 8) median survival 24.5 days, WT/BCR-ABL (*n* = 9) median survival 35 days. WT MIG (*n* = 4) and *Egr1*^-/-^ MIG (*n* = 4). Animals transplanted with MIGR1-infected BM did not show any signs of leukemia and were euthanized at the end of the experiments. **B.** Most leukemic mice exhibited myeloid disease. GFP+ BM cells harvested from leukemic mice when they exhibited signs of leukemia were prepared for antibody treatment, and incubated with anti-mouse Gr-1 and B220 antibodies. Following Facs, data analysis was performed using FloJo software. (Although all gates were not drawn at the 10^2^ positions on the y-axis, the cell populations are clearly observed and this does not impede the interpretation of the data). Eight to ten mice were analyzed from each genotype, and most of them (75-80%) developed myeloid leukemia. **C.** Representatives spleens of mice transplanted with either WT/BCR-ABL or *Egr1*^-/-^/BCR-ABL BM cells at 18 days post-BMT. Spleens were removed from mice transplanted with either WT/BCR-ABL or *Egr1*^-/-^/BCR-ABL BM cells following euthanasia at 18 days post-BMT.

**Figure 3:**
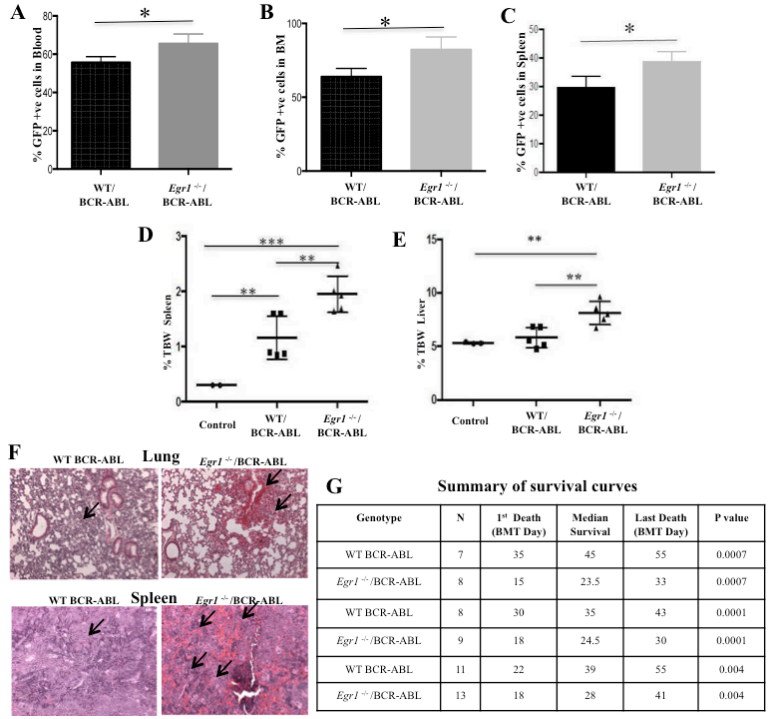
Loss of *Egr1* accelerated CML development in mice Lethally irradiated WT recipient mice were transplanted with BM from WT or *Egr1*^-/-^ mice infected with BCR-ABL-containing retrovirus as detailed above. For panels **A.**, **B.** and **C.**, mice were euthanized when moribund. Facs analysis was done on peripheral blood (A), BM (B) and spleen cells (C) to assess % GFP^+^ cells. (A), *P* value = 0.0442; (B), *P* value = 0.0370; (C), *P* value = 0.0442. WT/BCR-ABL *n* = 6, *Egr1*^-/-^/BCR-ABL *n* = 5. **D.** & **E.** Mice were euthanized 20 days after BMT, and spleen, liver and total body weight were determined. Mice transplanted with MIGR1-infected BM were used as controls. Values plotted represent the ratio of spleen or liver weight to total body weight (TBW). (D) Analysis of spleen weight. Spleens of *Egr1*^-/-^/BCR-ABL -transplanted mice were significantly larger than in controls and in WT/BCR-ABL-transplanted mice. %TBW spleen: *Egr1*^-/-^/BCR-ABL vs. WT/BCR-ABL- *p* value = 0.008; WT/BCR-ABL vs. Control - *p* value = 0.0075; *Egr1*^-/-^/BCR-ABL vs. Control - *p* value = 0.0002. (E) Analysis of liver weight. Livers of *Egr1*^-/-^/BCR-ABL -transplanted mice were significantly larger than those of animals transplanted with WT/BCR-ABL BM cells. %TBW liver: *Egr1*^-/-^/BCR-ABL vs. WT/BCR-ABL - significant, *p* value = 0.0080; WT/BCR-ABL vs. Control - not significant, *p* value = 0.4163; *Egr1*^-/-^/BCR-ABL vs. Control - Significant, *p* value = 0.0053. Control (*n* = 3), WT/BCR-ABL (*n* = 5), *Egr1*^-/-^/BCR-ABL (*n* = 5); **F.** H&E stained slides obtained from organs of leukemic mice showed more extensive infiltration of leukemic cells in *Egr1*^-/-^/BCR-ABL BM-transplanted mice compared to WT counterparts, in spleen and lungs. No clear infiltration of leukemic cells in the livers in either of the genotypes was observed. Five slides from organs from 3 mice for each genotype were examined; photograph is representative of what was observed for each genotype. Mice were displaying signs of leukemia when euthanized. Arrows indicate infiltration of leukemic cells. (10 × magnification). **G.** Summary of survival curves. Kaplan-Meier survival curves for recipients of MIGR1 or MIG BCR-ABL-transduced BM cells from WT or *Egr1*^-/-^ donor mice.

In order to determine the effect of loss of *Egr1* on the initiation and progression of CML, we used a mouse model of CML (Figure 1). Specifically we transplanted lethally irradiated WT recipient mice with BM from WT or *Egr1*^-/-^ mice infected with BCR-ABL-containing retrovirus (MSCV-IRES-GFP-BCR-ABL [MIGR1-BCR-ABL]). We observed that all the mice transplanted with BCR-ABL-expressing BM null for *Egr1* succumbed to leukemia significantly faster than those mice transplanted with BCR-ABL expressing BM WT for *Egr1* (*P* value = 0.0001) (Figure 2A and 3G). We next inquired if there is a difference in the type of leukemia, and observed that regardless of the genotype of the donor BM most animals developed myeloid leukemia, with GFP+ BM cells from leukemic mice expressing Gr1 and not B220 (Figure 2B). Not only did mice display more rapid onset of leukemia (Figure 2A), at the time when mice were in a moribund state the disease was more severe in mice transplanted with *Egr1*^-/-^/BCR-ABL BM than with WT/BCR-ABL BM. This can be seen in tissue sections from spleens and lungs, where there was more pronounced infiltration of leukemia cells in *Egr1*^-/-^/BCR-ABL BM recipients than the WT/BCR-ABL BM recipients (Figure 3F). Furthermore, more leukemic cells (GFP+) were observed in the blood, BM and spleens (Figure 3A-3C) in mice transplanted with *Egr1*^-/-^/BCR-ABL BM compared to WT/BCR-ABL BM. Consistent with these findings, there is a greater increase in spleen and liver size in mice transplanted with *Egr1*^-/-^/BCR-ABL BM compared to WT/BCR-ABL BM (Figure 3D-3E). Taken together these data demonstrate that Egr1 behaves as a tumor suppressor in CML.

### *Egr1* expression is down-regulated by BCR-ABL

**Figure 4:**
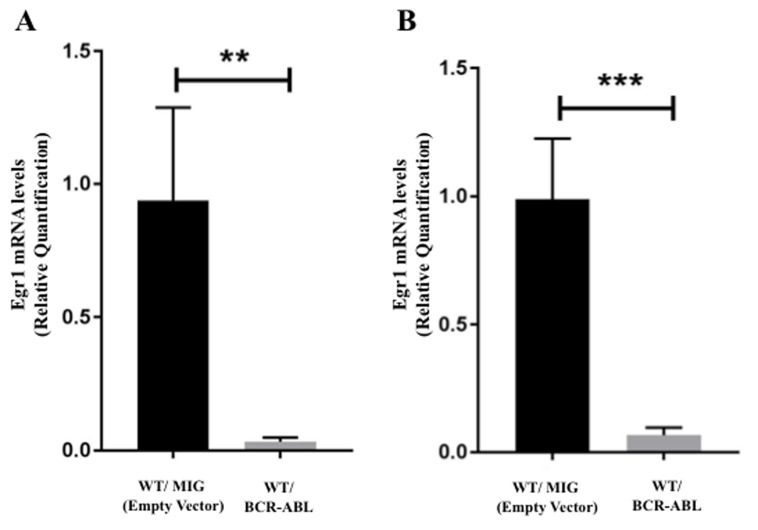
*Egr1* expression is down-regulated by BCR-ABL **A.** WT BM from 5-FU treated mice was transduced with either MSCV-BCR-ABL-IRES-GFP (MIG BCR-ABL) or the MSCV-IRES-GFP (MIGR1) retroviral vector. GFP^+^ cells were selected by cell sorting, expanded, and RNA extracted. Total RNA was analyzed by real time PCR for *Egr1* expression using taqman probe. 18S rRNA probe was used as an internal control. Each sample comes from a different mouse. WT/MIG *n* = 4, WT/BCR-ABL *n* = 5. **B.** Spleens were harvested from mice transplanted with WT BM infected with either MIGR1 vector (control) or MIG BCR-ABL vector, 20 days post-BMT. Cells from whole spleens were dissociated and red blood cells removed prior to RNA extraction. WT/MIG *n* = 3, WT/BCR-ABL *n* = 8.

Given the evidence that *Egr1* has tumor suppressor functions, we wanted to assess how its expression is regulated by the BCR-ABL oncogene. Using RNA from WT BM expressing BCR-ABL and empty vector control, a decrease in the level of *Egr1* mRNA in BCR-ABL expressing BM was observed (Figure 4A). The down-regulation of *Egr1* by BCR-ABL was further confirmed *in vivo* in mice 20 days post-BMT. Spleens obtained from mice transplanted with BCR-ABL-expressing BM showed reduced expression of *Egr1* when compared to empty vector controls (Figure 4B). These results demonstrate that BCR-ABL down-regulated *Egr1* expression, either directly or indirectly, in both BM cells *in vitro*, and in the spleens of transplanted mice. It should be pointed out that although *Egr1* expression is reduced in BCR-ABL expressing hematopoietic cells, loss of *Egr1*, similar to other critical tumor suppressors like *Pten* and and p53 [18, 25], still has a significant impact on the progression of BCR-ABL induced leukemia.

### *Egr1 deficiency* is associated with decreased apoptosis, and increased cell viability and proliferation in response to BCR-ABL

**Figure 5:**
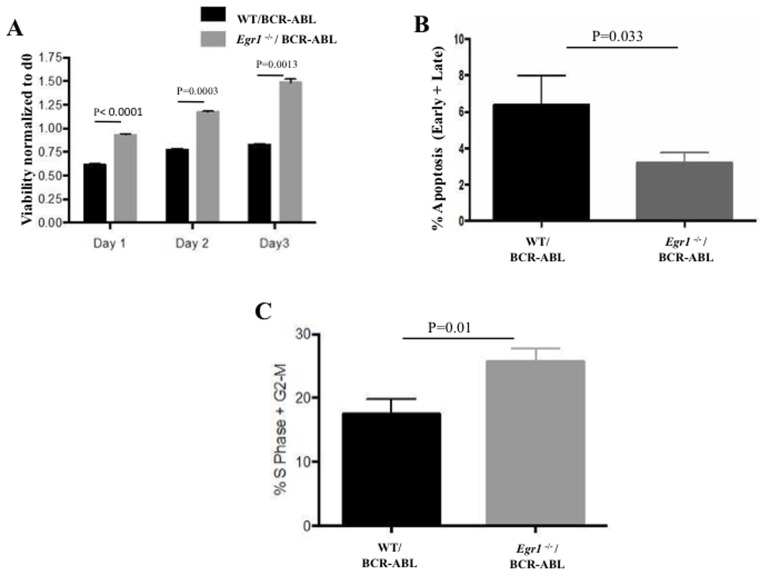
*Egr1 deficiency* is associated with decreased apoptosis, and increased cell viability and proliferation in response to BCR-ABL For **A.** and **B.**, *Egr1*^-/-^ and WT BM cells were infected with BCR-ABL expressing vectors, and GFP+ cells were sorted and used for analysis. Viability was assessed using MTS assay by normalizing to day 0. BCR-ABL infected *Egr1*^-/-^ BM cells showed enhanced viability at the three time-points compared to BCR-ABL infected WT BM cells. Day 1 - *P* value < 0.0001; Day 2 - *P* value = 0.0003; Day 3 - *P* value = 0.0013. Data is representative of five independent experiments. (B) GFP+ sorted BM cells were incubated with Annexin V antibody and PI, and Facs analysis was done. BCR-ABL infected *Egr1*^-/-^ BM have fewer Annexin V positive cells, thus fewer apoptotic cells, than BCR-ABL infected WT BM, *P* value = 0.033. Represented here are the percentage of early (Annexin V^+^ only) and late (Annexin V^+^ and PI^+^) apoptosis. Data is representative of 3 independent experiments. **C.** 20 days following BMT, mice were injected intraperitoneally with BrdU and euthanized after two hours. BM was harvested and prepared for cell proliferation assessment using BrdUassay. *P* value = 0.01. WT/BCR-ABL: G1-55.3%, S-8.3%, G2-M-11.1%; *Egr1*^-/-^/BCR-ABL: G1-60.3%, S-15.2%, G2-M-12.9%. Data is representative of 3 independent experiments. WT/BCR-ABL *n* = 3, *Egr1*^-/-^/BCR-ABL *n* = 4.

In an attempt to decipher how loss of *Egr1* accelerated the initiation and progression of CML, we investigated how its loss impacted on the survival and proliferation of BCR-ABL expressing BM cells. The change in viable cell number over time was ascertained using the MTS assay. It can be seen that *Egr1*^-/-^/BCR-ABL BM had increased number of viable cells compared to WT/BCR-ABL BM cells, and this increase in cell number was more rapid (Figure 5A). To determine if this was due to altered apoptosis, cell proliferation, or a combination of both, apoptosis and proliferation were assessed. *Egr1*^-/-^/BCR-ABL BM consistently showed a lower percentage of Annexin V positive cells, thus fewer apoptotic cells compared to WT/BCR-ABL BM (Figure 5B). In order to measure the rate of cell proliferation *in vivo*, mice transplanted with *Egr1*^-/-^ or WT/BCR-ABL BM were injected intraperitoneally with BrdU 20 days following BMT and assayed for BrdU incorporation. It was observed that BM from animals transplanted with *Egr1*^-/-^/BCR-ABL BM had an increased rate of DNA synthesis compared to BM from mice transplanted with WT/BCR-ABL BM, as shown by increased percentage of cells in S and G2-M phases (Figure 5C). Taken together, the decreased apoptosis and increased proliferation seen in *Egr1*^-/-^/BCR-ABL BM is consistent with its increased leukemic potential.

### *Egr1*^-/-^/BCR-ABL BM transplanted mice are enriched in leukemia stem cells (LSCs) compared to WT counterparts

**Figure 6:**
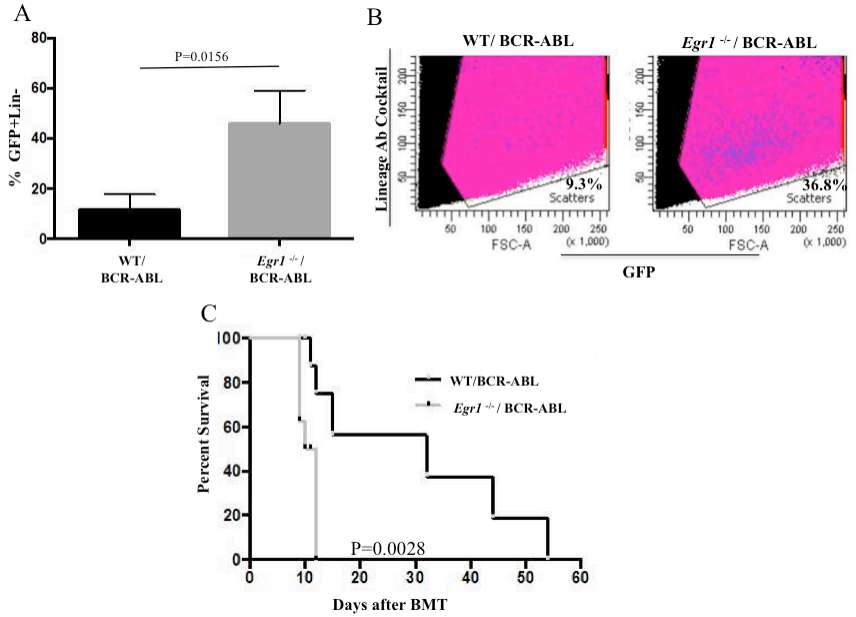
Loss of *Egr1* increased leukemia initiating cells **A.-B.** The leukemia stem cells are enriched in BM from mice transplanted with *Egr1*^-/-^/BCR-ABL BM compared to WT/BCR-ABL BM. BM was harvested 20 days post BMT. Representative Facs data of GFP^+^Lin^-^ cells from one of the mice analyzed for each genotype. *P* value = 0.0156. (*n* = 3 for each genotype) **C.** BM from primary transplanted mice was harvested on day 14 post BMT and red blood cells were lysed. 1x10^6^ BM cells from primary transplanted mice were injected into lethally irradiated secondary recipient mice, and Kaplan-Meier survival curves for recipients are shown. Statistical analysis was performed using Log-Rank (Mantel-Cox) test. *P* value = 0.0028. Median Survival: WT/BCR-ABL 32 days; *Egr1*^-/-^/BCR-ABL 11 days. WT/BCR-ABL (*n* = 6) and *Egr1*^-/-^/BCR-ABL (*n* = 7).

CML is a stem cell-derived disease and the accelerated development of CML due to *Egr1* deficiency (Figure 2 & 3) prompted us to examine how the loss of *Egr1* impacted leukemic stem/initiating cells (LSCs). First it was asked whether there was any quantitative difference in the stem cell population of mice transplanted with *Egr1*^-/-^/BCR-ABL BM compared to the WT/BCR-ABL BM transplanted mice. BM was harvested from mice 20 days post-transplantation and the percentage of GFP+, Lin- cells was determined by FACS analysis. Our results showed that there was a significant (*P* = 0.0156) increase in the percent of GFP^+^ Lin^-^ cells from animals receiving *Egr1*^-/-^/BCR-ABL BM (Figure 6A-6B). These results suggest a more rapid expansion of leukemic cells (GFP^+^ Lin^-^) in *Egr1*^-/-^/BCR-ABL BM recipients.

Next, we set out to investigate if there is a difference in leukemia initiating cells by testing the ability of *Egr1*^-/-^/BCR-ABL BM to serially transfer leukemia in mice. WT or *Egr1*^-/-^ BM cells were transduced with BCR-ABL and transplanted into lethally irradiated mice; 14 days post-transplantation BM harvested from primary transplanted mice was transplanted into lethally irradiated secondary recipient mice. All *Egr1*^-/-^/BCR-ABL recipient mice died by day 13 post-transplantation with a median survival of 11 days compared to WT/BCR-ABL recipient mice (median survival of 32 days) (Figure 6C), indicating that loss of *Egr1* resulted in enrichment of LSCs in the primary transplanted mouse. This is consistent with shorter survival time and more aggressive disease of these mice compared to WT counterparts.

### Loss of *Egr1* increased self-renewal ability of BCR-ABL expressing BM

**Figure 7:**
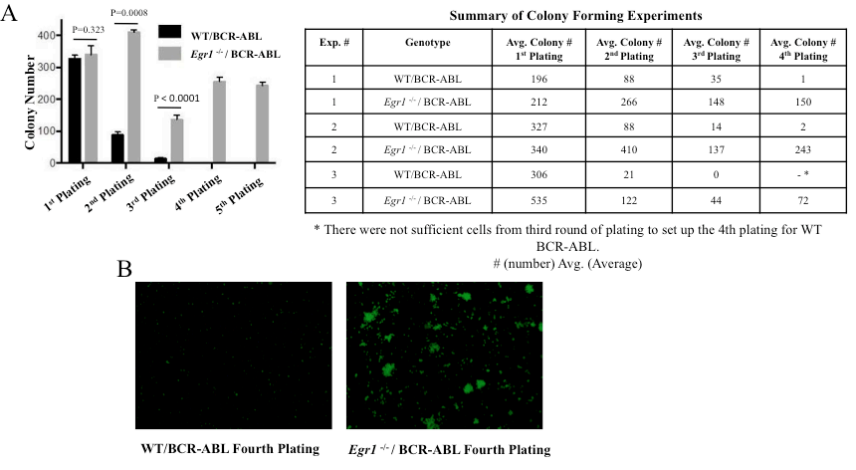
Loss of *Egr1* enhanced self-renewal capacity of BCR-ABL expressing BM GFP^+^ cells (WT/BCR-ABL and *Egr1*^-/-^/BCR-ABL) were plated for colonies, 8-12 days later colonies were counted and plated for the next round of colonies. **A.** Data from one representative experiment (experiment 2 shown in Table). Statistical analysis: first round, *P* value = 0.3232 not significant; second round: *P* value = 0.0008, third round: *P* value < 0.0001; fourth round: *P* value < 0.0001. Colony number is average number of colonies obtained from three 35mm dishes. **B.** Photomicrograph of representative plate from 4^th^ round of plating, 4 × magnification.

To ascertain if loss of *Egr1* impacts on the self-renewal properties of BCR-ABL expressing BM, serial re-plating colony assays were performed. GFP^+^ cells (WT/BCR-ABL and *Egr1*^-/-^/BCR-ABL) were plated for colonies, 8-12 days later colonies were counted and plated for the next round of colonies. Although there was no significant difference in the number of colonies for BCR-ABL expressing WT and *Egr1*^-/-^ BM in the first round of plating, the *Egr1*^-/-^ colonies were larger than WT. In the second round there was a significant reduction in the WT colony numbers in contrast to *Egr1*^-/-^ cells. Furthermore, *Egr1*^-/-^/BCR-ABL was serially passaged at least five times without losing its ability to form colonies, whereas for WT/BCR-ABL cells colony numbers continued to be reduced with very few colonies by the third passage (Figure 7A). In addition, the colonies formed by *Egr1*^-/-^/BCR-ABL BM cells continued to be much larger than the colonies formed by WT/BCR-ABL cells (Figure 7B). These data demonstrate that loss of *Egr1* increased self-renewal ability in BCR-ABL-expressing BM.

**Figure 8:**
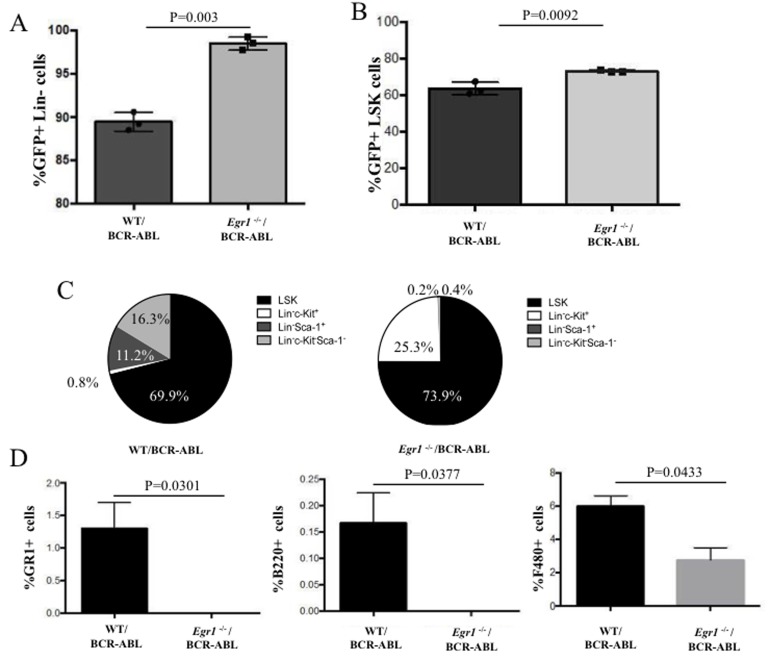
Immuno-phenotype of cells from colonies from BCR-ABL expressing WT- and Egr1^-/-^ BM After counting colony numbers on passage 3, cells were re-suspended in PBS and washed to remove methylcellulose; then stained with the indicated antibodies for Facs analysis. **A.** Percent of total cells that are GFP+, Lin-. *P* value = 0.0003. **B.** Percent of total cells that are GFP^+^ and LSK. *P* value = 0.0092. **C.** Distribution of Lin- cell population for Sca-1 and c-Kit expression. **D.** Cells from WT/BCR-ABL colonies have higher number of cells expressing lineage markers (Gr-1, B220 and F4/80). Statistical analysis: Gr-1 *P* value = 0.0301; B220: *P* value = 0.0377; F-480 *P* = value 0.0433.

Finally, to understand why loss of *Egr1* allowed for continuous colony formation for at least several passages, the immunophenotype of the cells comprising the colonies was determined. Specifically, the frequency and distribution of immature cell types, as well as the presence of differentiated cells was compared using cells from WT and *Egr1*^-/-^ colonies taken after three passages. It should be kept in mind that there were substantially fewer colonies in WT than Egr1^-/-^ cells, and colony formation selects for proliferating cells. In spite of this, we observed that there were a higher percentage of GFP^+^ Lin- and Lin^-^ Sca-1^+^c-Kit^+^ (LSK) cells in the colonies formed by *Egr1*^-/-^/BCR-ABL BM than in the WT/BCR-ABL BM colonies (Figure 8A-8B). Interestingly, it was observed that the colonies formed by *Egr1*^-/-^/BCR-ABL cells had a more homogeneous population than the WT counterpart, with most of its cells being LSK (73.9%) and Lin^-^ Sca-1^-^c-Kit^+^ (25.3%), therefore 99.2% of cells were both lineage negative and positive for c-Kit (Figure 8C). In contrast, very few WT cells were Lin-Sca1-cKit+. In addition, all differentiation markers were higher in WT than *Egr1*^-/-^/BCR-ABL -expressing cells (Figure 8D). Taken together, these data show that *Egr1*^-/-^/BCR-ABL BM has higher self-renewal ability than WT/BCR-ABL BM, and is accompanied by an increase in Lin-Kit+ cells.

## DISCUSSION

To study the role of *Egr1* as a tumor suppressor of myeloid leukemia, a mouse model of BCR-ABL driven leukemia was employed. We have shown that loss of *Egr1* accelerated the development of leukemia in the bone marrow transplantation and transduction model of CML. Furthermore, loss of *Egr1* is associated with increased numbers of LSCs, which have enhanced ability to self-renew. Most of the transplanted mice developed a CML-like disease, while a smaller group developed a B cell lymphoblastic leukemia [B-ALL]-like disease (data not shown). The two diseases observed in our BM transplantation have been reported in other laboratories [26-28]; the incidence of B-ALL-like leukemia in both WT and *Egr1*^-/-^/BCR-ABL induced leukemia may be mouse strain specific.

The BM from *Egr1*^-/-^/BCR-ABL leukemic mice were enriched with lineage negative BCR-ABL-expressing cells, compared to WT counterparts (Figure 6A-6B), supporting a role for loss of *Egr1* increasing the LSC population. Min et at.[29] observed that *Egr1*^-/-^ HSCs cycle more frequently than WT; however, the excess HSCs produced do not remain in the BM but rather leave the BM niche and enter the blood stream increasing the number of circulating HSCs [29]. It is possible that loss of *Egr1* alters the homeostasis of LSCs, similar to its effect on HSCs, by increasing the population of cycling cells, thereby accounting for the observed early and consistent elevation of LSCs in leukemic mice. Consistent with these observations, loss of *Egr1* significantly enhanced the self-renewal capacity of BCR-ABL-expressing BM. Whereas the colony replating of WT/BCR-ABL-expressing BM declines rapidly with few or no colonies by the third cycle, loss of *Egr1* was associated with continued colony formation at least until the fifth cycle, with no sign of abating (Figure 7). In addition, the cells comprising the *Egr1*^-/-^ colonies were more homogenous than WT, enriched for LSK cells, with the remainder being c-Kit positive; 99.2% of cells were both lineage negative and positive for c-Kit (Figure 8C).

The role of *Egr1* as a positive regulator of myeloid differentiation, as well as its role in HSC homeostasis, could, in combination, participate in the observed increase in primitive cells and lack of differentiated cells in *Egr1*^-/-^/BCR-ABL expressing cells. These observations are consistent with the loss of *Egr1* resulting in more rapid onset and more aggressive leukemia, and the increased burden of lineage negative cells in the BM of BCR-ABL-induced leukemia. The effect of serial passaging of *Egr1*^-/-^/BCR-ABL expressing cells, as done in colony assays, on their ability to promote leukemia compared to non-passaged cells would be informative. Furthermore, within that context how the gene expression signature is altered in association with passaging would inform us on how loss of *Egr1* interacts with different signaling pathways to modify the leukemic phenotype and how it changes over time.

Similar to other critical tumor suppressors like *Pten* and *Msr1* [18, 36], *it was observed that Egr1* expression is down-regulated by BCR-ABL both *in vitro* as well as *in vivo* (Figure 4). This is consistent with its tumor suppressor role; failure to reduce *Egr1* may limit or inhibit leukemia initiation and progression. As reported in this manuscript, total loss of *Egr1* allows the leukemia to be initiated and to progress more rapidly.

EGR1 has been shown to regulate cell growth, proliferation, and apoptosis in different cell types by engaging key signaling pathways such as TGFβ1, PTEN, p53 and fibronectin [17, 31, 34]. It will therefore be interesting to study their functional hierarchy and relative contribution in CML by performing pharmacological inhibitor studies.

Previous reports have revealed an important role for Egr1 in different hematological conditions: Egr1 regulates hematopoietic stem cell proliferation and localization [13]; haploinsufficiency of Egr1 promotes N-ethyl-nitrosourea (ENU) induced myeloproliferative disorder (MPD) [30]; concordant loss of Tp53 in Egr1 and Apc haploinsufficient HSPCs leads to AML in mice [31]; miRNA-181a modulates acute lymphoblastic leukemia by targeting Egr1[32]; miRNA-146a modulates B-cell oncogenesis through Egr1 [33]; and the NF-κB/EGR1/BIM pathway regulates cytotoxicity of mTOR dual inhibitors in malignant lymphoid cells[34]. Our data provide an important extension of this notion, showing for the first time that loss of *Egr1* accelerates BCR-ABL driven chronic myelogenous leukemia.

The studies on Egr1 in CML provide the impetus to study the effect of altering Egr1 expression in AML where the overall five year survival rate remains low. The effect of loss of Egr1 in CML could reflect its established functions in normal hematopoiesis, maintaining quiescence of HSCs and driving terminal differentiation to the monocyte/macrophage lineage. Gain of function studies should validate these conclusions and provide further rationale for studying the effect of increased Egr1 as a therapeutic target in AML.

In conclusion, the results obtained indicate that *Egr1* is a critical tumor suppressor of BCR-ABL driven chronic myelogenous leukemia providing the impetus to further investigate the role of *Egr1 and explore its potential as a therapeutic target in other cancers.*

## MATERIALS AND METHODS

### Cell lines and primary bone marrow cells

Phenix Ecotropic (Eco) and Gryphon Ecotropic (Eco) (purchased from Allele Biotech) retrovirus packaging cell lines were used to generate retrovirus. Both cell lines were created by placing constructs capable of producing gag-pol and envelop proteins for ecotropic viruses into 293T cells. Cells were cultured in Dulbecco’s modified Eagle’s medium (DMEM) (Corning Cellgro) supplemented with 10% fetal bovine serum (FBS) (Gibco) and 100 U/ml penicillin, 100 μg/ml streptomycin (Corning CellGro). Cell culture was carried on a humidified tissue culture incubator with 5% CO_2_ at 37°C. Primary bone marrow (BM) cells were harvested from C57BL6 mice and *Egr1*^-/-^ C57BL6 mice injected with 200mg/g of 5-Fluourouracil (5-FU) 4-5 days prior to BM harvest. After BM harvest, the red blood cells were lysed with Ammonium Chloride Potassium (ACK) lysing buffer (Gibco), cell pellet was washed with phosphate buffered saline (PBS) (Gibco Cellgro) and the white blood cells were then cultured in Iscove’s modified Dulbecco’s medium (IMDM) (Corning Cellgro) supplemented with 10% FBS, 100 U/ml penicillin, 100 μg/ml streptomycin and the cytokines Stem Cell Factor (SCF) (50 ng/ml), Interleukin-6 (Il-6) 15 (ng/ml) and Interleukin-3 (Il-3) (10 ng/ml) (Peprotech). Cell culture was maintained in a humidified tissue culture incubator with 10% CO_2_ at 37°C.

### Mice

C57BL6 mice and *Egr1*^-/-^ C57BL6 mice were obtained from Michelle LeBeau (University of Chicago) with permission from J. Millbrandt (Washington University School of Medicine in Saint Louis), who established the strain [35]. Mice were maintained in a temperature and humidity-controlled environment at Temple University’s Health Science campus animal facilities. The Temple University Institutional Animal Care and Use Committee (IACUC) approved all animal studies. Since *Egr1*^-/-^ female mice are sterile breeding for generation of *Egr1*^-/-^ mice was done using *Egr1*^-/-^ male mice with *Egr1* ^+/-^ females. Pups were ear-tagged and tails clipped at the time of weaning. Tail clippings used for genotyping, were put in lysis buffer solution (50μl) overnight in 55°C water bath. Afterwards 300 μl of DNase free water was added to the tubes containing individual lysed tail clippings and boiled for 5 minutes. Once cooled this mix of tail, buffer and water was used as DNA template for PCR reactions to determine the presence or absence of the *Egr1* gene. Primers used were: *Egr1* WT R - 5’-ggg cac agg gga tgg gaa tg-3’; *Egr1* WT F - 5’-aac cgg ccc agc aag aca cc-3’; *Egr1* Neo F - 5’- ctc gtg ctt tac ggt atc gc-3’. All three primers have to be in each PCR reaction to observe *Egr1* status on the sample.

### Bone marrow transduction and transplantation

Bone marrow cells were harvested from mice that were injected with 200mg/g of 5-FU intra-peritoneally 4-5 days prior to BM harvest. 48 hours after harvest, cells were subjected to 2-4 rounds of spin infections with retroviral supernatant carrying MSCV-IRES-GFP (also called MIGR1) or MSCV-IRES-BCR-ABL-GFP (also called MIG BCR-ABL) (generated using Phoenix Eco or Gryphon Eco cells), in the presence of polybrene (Sigma)(10mg/ml) and cytokines (SCF 20ng/ml, Il-3 and Il-6 8 ng/ml). 24 hours after the last round of infections, cells were analyzed on BD FACS Calibur to determine the efficiency of infection (percentage of GFP^+^ cells). Retro-orbital injection of 3 × 10^3^ GFP^+^ cells and 4.97x10^5^ uninfected cells was done into each lethally irradiated WT recipient mice (1 irradiation dose of 900 Rads using a RS-2000 Biological Irradiator) (Rad Source). Secondary BM transplantation was done using BM from primary BM transplanted mice. After removing red blood cells with ACK lysis, cells were washed with PBS and centrifuged at 1000 RPM for 5 minutes; then, the cell pellet was resuspended in PBS. 1x10^6^ white blood cells were injected into each lethally irradiated secondary recipient mice by retro orbital injection[36].

### Flow cytometric analysis

BM and spleen cells were dissociated and treated with ACK lysis buffer to lyse the red blood cells, washed with PBS and suspended in PBS + 1% BSA (FACS buffer). Antibody staining was performed on ice for 30 minutes unless specified by the maker. Flow cytometric analysis was carried in a BD FACS Calibur or a BD LSR II flow cytometer as described previously [37]. Data analysis was performed using FACS Diva (BD Biosciences) or FlowJo software.

### Antibodies

PerCP-Cy 5.5 Mouse lineage cocktail, APC c-Kit, APC Gr-1, PE B220, APC anti-BrdU and Fc block (CD16/CD32) were all from BD Pharmingen. Pacific blue anti-mouse Ly-6A/E (Sca-1) was purchased from Biolegend. PE F4/80, APC CD11b and APC Annexin V were from eBioscience.

### Analysis of cell viability

Sorted GFP^+^ BM cells expressing either MSCV-IRES-GFP or MSCV-IRES-BCR-ABL-GFP were cultured in IMDM supplemented with 10% FBS, 100 U/ml penicillin, 100 μg/ml streptomycin and the cytokines SCF (50 ng/ml), Il-6 (15 ng/ml) and Il-3 (10 ng/ml). 3-7 days post-sorting cells were plated in 96 wells plate at a density of 1x10^4^ cells/well. Afterwards at the 0, 24, 48 and 72 hours time points 20 μl of Cell Titer 96^®^ Aqueous One Solution Cell Proliferation Assay (Promega) were added to each well. Plates were incubated for 1-4 hours in a humidified 5% CO_2_ tissue culture incubator, according to manufacture’s protocol. The absorbance was then recorded at 490nm using a 96-well Victor plate reader (Perkin Elmer).

### Analysis of apoptosis

Cell apoptosis was measured using Annexin V- APC apoptosis detection kit along with Propidium Iodide (PI) staining solution according to the manufacturer instructions (eBioscience). WT/BCR-ABL and *Egr1*^-/-^/BCR-ABL infected BM cells were washed in PBS once then washed in binding buffer supplied in the kit. Cells were incubated with Annexin V antibody for 10-15 minutes at room temperature, washed with wash buffer supplied in the kit and resuspended in binding buffer, at this point 5μ of PI solution was added to the cells. Flow cytometric analysis of cells was performed within 2 hours of staining using FACSCalibur or LSR II, and data was analyzed on either FlowJo or FACSDiva software.

### Analysis of cell proliferation using bromodeoxyuridine (BrdU) assay

Mice transplanted with WT/BCR-ABL and *Egr1*^-/-^/BCR-ABL were intraperitoneally injected (IP) with 2mg of BrdU solution (10 mg/ml solution of BrdU in 1 × DPBS). 2 hours after injection, mice were euthanized and bone marrow was harvested. Staining was performed according to manufacturer’s protocol (BD Pharmingen). Briefly, cells were fixed and permeabilized with BD Cytofix/Cytosperm buffer for 30 minutes on ice, then washed with 1 × BD Perm/Wash buffer. Afterwards cells were incubated on ice for 10 minutes with BD Cytosperm Permeabilization Plus and washed again with wash buffer. Cells were then re-fixed for 5 minutes on ice with the fixing buffer, and again washed before being treated with DNase for 1 hour at 37°C. After DNase treatment cells were once more washed in wash buffer and incubated with APC conjugated anti-BrdU antibody for 20 minutes at room temperature. Following incubation with the antibody, cells were washed in wash buffer one last time and suspended in PI solution. Stained cells were acquired on LSR II and analyzed using FACS Diva software.

### Serial re-plating colony assay

WT and *Egr1*^-/-^ BM expressing BCR-ABL were obtained following infection of appropriate BM with the MSCV-IRES-BCR-ABL-GFP vector, as described in section “Bone Marrow Transduction and Transplantation” followed by sorting to select for GFP+ expression. The cells were suspended in IMDM with 2% FBS at a concentration of 2 × 10^5^ cells/ml. Then, 0.4 ml of this cell suspension was added to 4 ml of MethoCult (StemCell Technologies). Cells were mixed thoroughly and 1.1ml of MethoCult + cells was dispensed into each 35 mm dish. Final cell concentration was 2x10^4^ cells/dish. Dishes were incubated at 37°C, 5% CO_2_, with ≥95% humidity for 8-12 days to allow colonies to form. Colonies were then counted; the cells were rinsed with PBS, counted and replated in new media at a concentration of 2 × 10^4^ cells/dish to test their ability to form colonies upon secondary plating. This process was repeated as indicated for each experiment.

### RNA extraction and quantitative polymerase chain reaction

WT/BCR-ABL and *Egr1*^-/-^/BCR-ABL GFP sorted BM, as well as dissociated spleen cells from animals transplanted with WT/BCR-ABL or *Egr1*^-/-^/BCR-ABL BM, were washed in PBS, spun in a centrifuge at 1000 RPM for 5 minutes, then supernatant was removed and cell pellet frozen at -80°C freezer until RNA extraction. RNA was extracted from samples using RNaesy kit (Qiagen) according to manufacturer’s protocol. A reverse transcriptase polymerase chain reaction (RT-PCR) was performed to convert RNA to c-DNA using TaqMan Reverse Transcription reagents (Applied Byosistems) according to manufacturer’s protocol. c-DNA was then used to run the real time polymerase chain reaction analysis (qRT-PCR) in a StepOne Real Time PCR machine (Applied Byosistems) as described previously [38]. Probes used were the following, all purchased from Life Technologies: Mm00656724_m1 (mouse *Egr1*), and Mm04277571_S1, for 18, which was used as endogenous control.

### Tissue fixation and slide preparation

Spleens, liver and lungs were dissected from moribund mice and fixed in a 4% formaldehyde solution immediately. Slides from these fixed organs (prepared by Histotechnology Core, The Wistar Institute) were stained with Hematoxylin & Eosin (H&E) and examined using an Olympus inverted microscope with digital imaging.
